# Targeting murine leukemic stem cells by antibody functionalized mesoporous silica nanoparticles

**DOI:** 10.1038/s41598-017-18932-4

**Published:** 2018-01-17

**Authors:** Tamoghna Mandal, Michaela Beck, Nicole Kirsten, Mika Lindén, Christian Buske

**Affiliations:** 1 Institute for Experimental Cancer Research, CCC and University Hospital of Ulm, Albert-Einstein-Allee 11, 89081 Ulm, Germany; 2 Institute for Inorganic Chemistry II, University of Ulm, Albert-Einstein-Allee 11, 89081 Ulm, Germany

## Abstract

Acute leukemia is initiated and maintained by leukemia stem cells (LSCs) and therefore there is great interest to develop innovative therapeutic approaches which target LSCs. Here we show that mesoporous silica nanoparticles (MSNs) functionalized with succinic anhydride, tagged with an anti-B220 antibody and loaded with the anthracycline daunorubicin are efficiently incorporated into murine B220-positive AML LSCs and preferentially kill these cells in comparison to B220-negative AML LSCs *in vitro*. Furthermore, short – term treatment of the AML LSCs with these MSNs before transplant significantly delayed leukemia development in recipient mice. These data demonstrate that targeting of AML LSCs can be improved by using functionalized and antigen directed MSNs as carriers for anti-leukemic drugs.

## Introduction

One of the major goals in developing curative treatments in cancer is to target so called cancer stem cells (CSCs), which are responsible for initiating and maintaining tumor growth in many cancer subtypes. CSC targeting treatments should be able to eradicate the CSC without harming its normal counterpart, by this generating a therapeutic window allowing to treat the patient with acceptable therapy related toxicity^1^. One of the major obstacles for the development of such therapeutic concepts is the similarity between CSCs and their normal counterparts^2^. Thus, many surface antigens are shared between malignant and normal tissue stem cells and there is a larger overlap in molecular programs driving growth of CSCs and normal stem cells. One of the cancers obeying the CSC model is acute myeloid leukemia (AML). The cornerstone of therapy in AML is dose intense polychemotherapy, using drugs such as anthracyclines or cytarabine. This treatment approach is largely unchanged since the 1970s and improvement in treatment outcome is virtually lacking in elderly patients suffering from this disease^3,4^. Thus, developing innovative therapeutic concepts is a major goal for this disease, from which still up to eight of ten patients are dying today. Experiments in murine models as well as in xenograft models using primary human AML samples have convincingly proven that AML is propagated by leukemic stem cells (LSCs)^5,6^. We and others have shown that LSCs share many characteristics with normal hematopoietic stem cells (HSCs) with regard to their antigen profile, but also with regard to their transcriptional profile^7–10^. Differences in the antigen profile between LSCs and HSCs would open the door to antibody – based preferential targeting of LSCs, sparing normal HSCs. We previously showed that AML can be propagated by LSCs with lymphoid characteristics in a CALM-AF10 positive AML model. These LSCs showed incomplete immunoglobulin DJ rearrangement, but also importantly expressed the lymphoid associated antigen B220 in contrast to normal HSCs. We now provide evidence that a multifunctional particle system based on zwitterionic mesoporous silica nanoparticles (MSNs) functionalized with the B220 antibody and loaded with the anti-leukemic drug daunorubicin is able to target AML LSCs in this model. This is the first study which demonstrates that functionalized mesoporous silica nanoparticles can be used for targeted drug delivery to AML stem cells.

## Results

### Morphology and size of nanoparticles

**Figure 1:**
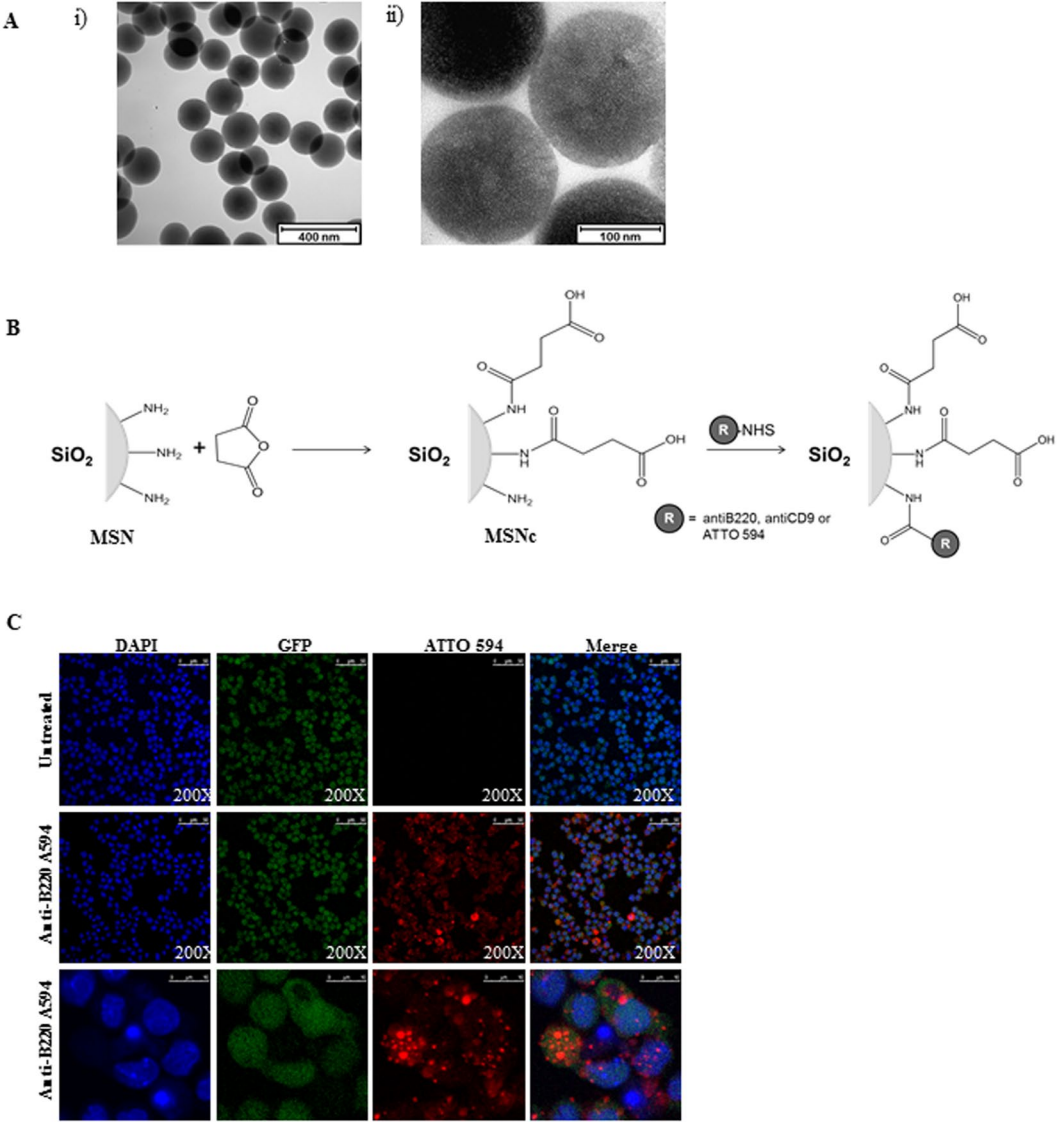
(**A**) Transmission electron microscopy images of MSN. Characterization of particles size and (i) morphology and (ii) analysis of pore structure. Scale bar is given. (**B**) Scheme of particle functionalization with succinic anhydride, antibody (anti-human/mouse-B220 (CD45R) or anti-human-CD9) and fluorescent dye (ATTO 594). (**C**) B220^+^ AML LSCs (CALM-AF10 cells) were treated with the anti-B220 tagged MSNs particles for 24 hours and spotted on glass slides and were visualized by confocal fluorescence microscopy. Nuclei were stained with DAPI (Blue), GFP (Green) is expressed retrovirally by the cells line and ATTO 594 (Red) was covalently linked to the MSNs. One representative image from three (n = 3) independently performed experiments. Particle concentration used is 50 µg/mL. For the upper and middle panel of images a magnification of 200X was used. The lower panel shows 4X zoomed in areas of the middle panel.

In a first step MSNs were generated suitable for targeting B220^+^ AML LSCs. Amino-functionalized MSNs were used as the starting particles^11^. Particle morphology and size (Fig. 1A,(i)), as well as mesopore structure (Fig. 1A,(ii)), were analyzed via transmission electron microscopy (TEM). The particles were spherical with a relatively narrow particle size distribution peaking at 190 nm (data not shown). Nitrogen sorption analysis confirmed a narrow pore size distribution peaking at 3.4 nm and a high surface area, 860 m²/g, in good agreement with published literature (Supplementary Table S1). In order to create a zwitterionic surface, the amino-groups were partially converted into carboxylic acid groups through succinic anhydride (0.4 mmol/g) treatment. For simplicity, these particles are also denoted as MSNs in the following (Fig. 1B). Successful surface functionalization was confirmed by zeta potential measurements. The amino-functionalized MSNs had a zeta potential of -9 mV under biologically relevant conditions (HEPES buffer solution, 25 mM, pH 7.2) while MSNs exhibited a more negative zeta potential of -27 mV under the same conditions as expected. The surface area of the MSNs particles was still high, 710 m²/g, and the mesopore diameter remained unchanged upon succinic anhydride treatment, showing that the treatment did not affect the structural properties of the particles. The MSNs were labelled with the red fluorescent dye ATTO 594 prior to antibody functionalization in order render them visible by optical methods during *in vitro* and *in vivo* experiments. To target the particles specifically to B220 positive murine AML LSCs in the CALM-AF10 model, a IgG2α, kappa monoclonal anti-B220 which reacts with the exon A-restricted isoform of the mouse CD45R was covalently conjugated to remaining amino groups on the MSNs through EDC/NHS-coupling. The amount of anti-B220 antibody on the particles was 7.6 ± 0.6 μg/mg MSNs, as determined through fluorescence spectroscopic analysis of the supernatant after the EDC/NHS coupling reaction coupling of fluorescent labelled anti-B220 and thorough washing. Particle uptake of fluorescently labelled anti-B220 tagged MSNs (anti-B220 MSNs) in B220^+^ murine AML LSCs *in vitro* was analyzed by confocal laser microscopy. Intracellular localization was proven by co-localization of the GFP protein, expressed by the retrovirally transduced CALM-AF10 positive AML LSCs and the ATTO 594 dye conjugated to the MSNs (Fig. 1C). To determine the extent of unspecific uptake of anti-B220 MSNs, B220 negative murine AML LSCs, derived from a murine AML model driven by the overexpression of the proto-oncogene Cdx2, were incubated with the anti-B220 MSNs for 24 hours before analysis on the fluorescence microscope. There was a major difference in uptake of the anti-B220 MSNs between the murine B220^+^ AML LSCs compared to the B220^-^ AML LSCs (Supplementary Fig. S1). Only few red speckles were visible in the B220^-^ murine AML LSCs, in line with non-specific uptake. Thus, these data demonstrate preferential uptake of MSNs into B220^+^ AML LSCs, when tagged with an anti-B220 antibody compared to leukemic cells lacking B220 receptor in abundance, B220^-^ AML LSCs.

### Drug loading and cytotoxicity

**Figure 2:**
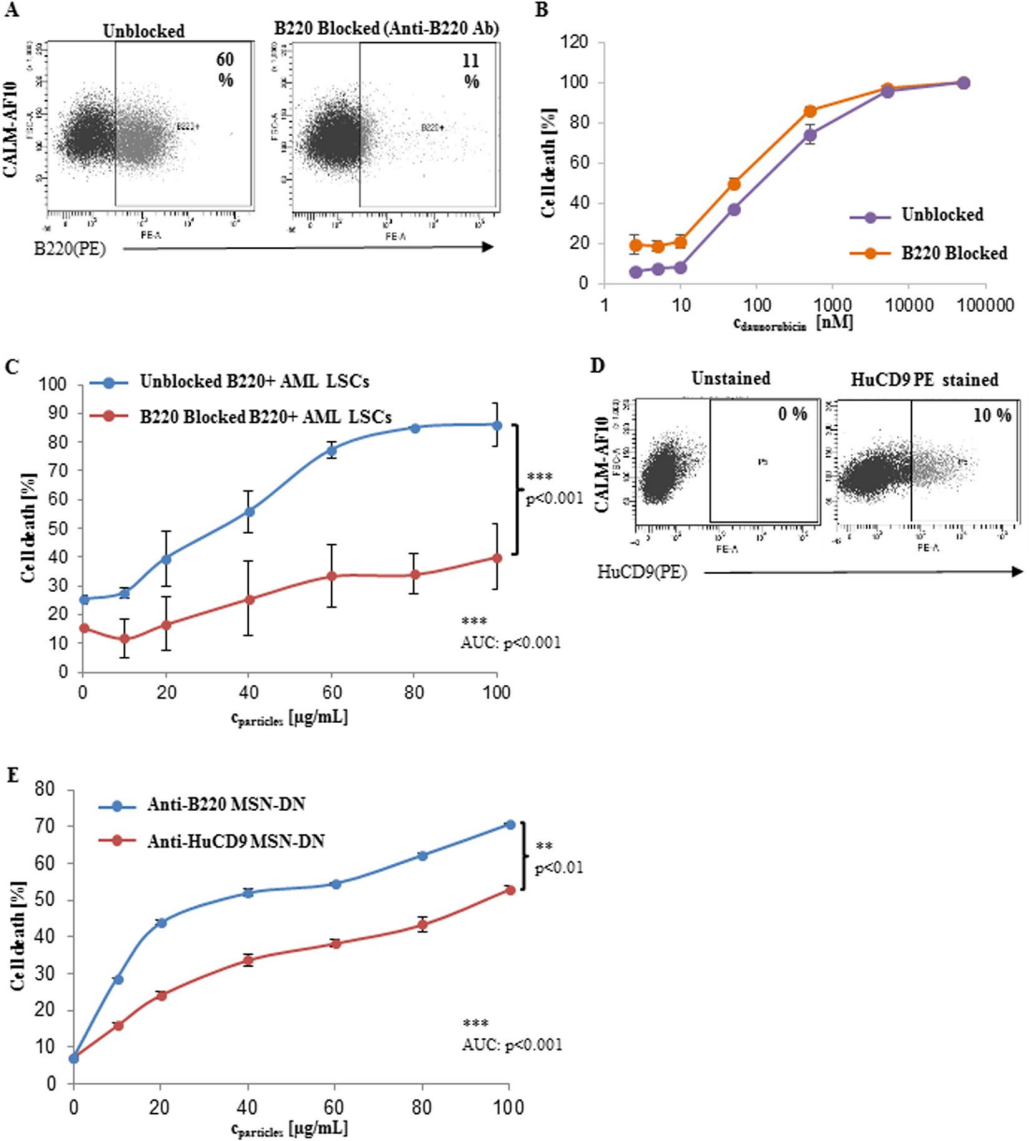
(**A**) FACS dot plot demonstrating B220 positivity of the B220^+^ AML LSCs after blockage with the unlabeled anti-B220 antibody for 4 hours and subsequent staining with the anti- B220-PE conjugated antibody compared to an unblocked control (n = 3). Cell death of B220^+^ AML LSCs blocked by the anti-B220 antibody after incubation with different concentrations of (**B**) free daunorubicin (n = 3) or (**C**) with anti-B220 MSN-DN compared to unblocked cells (n = 3) (p = n.s. and p < 0.001, respectively). (**D**) Representative FACS dot plot showing expression of the CD9 antigen on B220^+^ AML LSCs (CALM-AF10 cells)(n = 3). (**E**) Percent death of B220^+^ AML LSCs induced by daunorubicin loaded MSNs at different particle concentrations after 24 hours incubation as indicated (n = 3)(p < 0.01).

Daunorubicin (DN) is one of the most commonly used chemotherapeutics in the treatment of AML and as an anthracycline forms the backbone of polychemotherapy together with Ara-C^4,12^. For targeted drug delivery into B220^+^ AML LSCs, anti-B220 MSNs were loaded with DN in dichloromethane. DN loading was estimated by UV/vis spectroscopic analysis of starting, intermediate and final supernatants, prior to antibody functionalization. The amount of DN retained in the MSNs after conjugation with the anti-B220 antibody is shown along with the figure details in the table (Supplementary Table S2). To corroborate that cytotoxicity was due to active targeting of anti-B220 MSNs containing DN (MSN-DN) to B220^+^ AML LSCs, followed by cellular uptake and intracellular release of DN, B220 antigen was blocked on B220^+^ AML LSCs. Incubating B220^+^ AML LSCs with the unlabeled anti-B220 antibody for 4 hours resulted in 81.6% B220 antigen blockage, at 24 hours (Fig. 2A). Cell death induced by free DN did not significantly differ between B220 antigen blocked and unblocked cells (Fig. 2B). However, B220 antigen blocking significantly reduced cell death by anti-B220 MSN-DN by over 50% compared to unblocked control cells (mean of 40.2% ± 11.38 vs 86.05% ± 7.71, respectively, at a particle concentration of 100 µg/mL; p < 0.001) (Fig. 2C). The difference in cell death was also significant, across particle concentrations, ranging from 10, 20, 40, 60, 80 and 100 µg/mL, when cell death percentages were analyzed by the area under the curve method (AUC analysis) (p < 0.001) (Fig. 2C). In these experiments (Fig. 2B,C) both the concentration ranges are different, due to limitations corresponding to DN loading, so that the mean percentage cell death values was transformed into PROBIT values^13^ and plotted to estimate the incremental effect of DN when packed in the MSNs (Supplementary Fig. S2). Linear curve fitting and coefficient of determination (R^2^) values represented efficient data fitting for all the corresponding mean values. 24 hours treatment of anti-B220 MSN-DN on B220^+^ AML LSCs at lower drug concentrations achieved maximum killing in contrast to free DN, indicating better efficacy of DN loaded into functionalised MSNs (Supplementary Fig. S2).

To further verify specificity of the anti-B220 MSN-DN particles, MSNs conjugated to the anti-human CD9 antibody (anti-HuCD9) and loaded with DN were generated. This antibody crossreacts with murine CD9, which is expressed at low levels on the B220^+^ AML LSCs, mimicking the situation, in which the surface marker is present on both HSCs and LSCs, but at considerably different quantities. 10% of the B220 positive AML LSCs expressed CD9, whereas nearly all CD9 positive cells co-expressed B220 (Fig. 2D, Supplementary Fig. S3). There was a significant higher cell death between anti-B220 MSN-DN compared to anti-HuCD9 MSN-DN at all different particle concentrations with up to 1.3 fold (mean of 70.65% ± 0.6) increased cell death with the anti-B220 MSN-DN and anti-HuCD9 MSN-DN at highest particle concentration of 100 µg/ml (p < 0.001)(Fig. 2E). Significant and consistent difference across different particle concentrations was also observed between the anti-B220 MSN-DN and anti-HuCD9 MSN-DN treatment on B220^+^ AML LSCs (Fig. 2E).

### Impact on AML development *in vivo*

**Figure 3:**
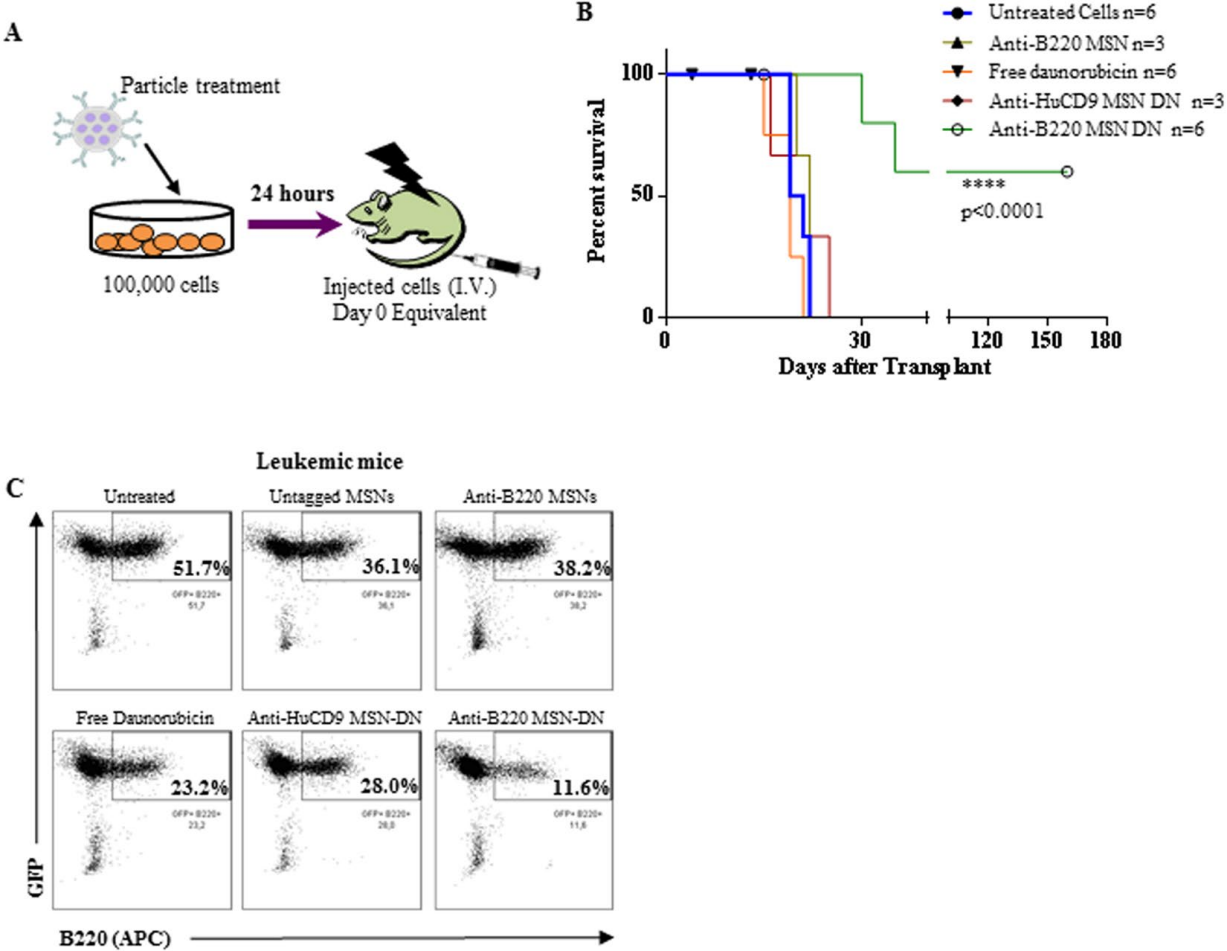
(**A**) Schematic diagram of the experimental set up of the experiments testing the impact of MSNs on the leukemia repopulating activity of B220^+^ AML cells. (**B**) Survival curve of mice transplanted with the day 0 equivalent of 1 × 10^5^ B220^+^ AML LSCs after 24 hours treatment with the different MSNs particles as indicated. Mice numbers originating from two independent experiments are shown (p < 0.0001). 3 of 6 mice treated with the anti-B220 MSN-DN particles stayed healthy until day 160 post-transplant (see Supplementary Fig. S4). (**C**) Dot plots of BM of representative mice succumbing to leukemia in the different experimental arms showing lowest proportion of LSCs (GFP^+^B220^+^) for the anti-B220 MSN-DN treatment arm.

To confirm the potential of the anti-B220 MSN-DN to impair AML LSC activity, B220^+^ AML LSCs were treated with either untagged MSNs, anti-B220 MSNs, free DN, anti-HuCD9 MSN-DN or anti-B220 MSN-DN for 24 hours. Equivalent cell numbers were subsequently injected into lethally irradiated recipients (Fig. 3A) (Supplementary Table S3). In these experiments particle concentrations were adjusted to achieve the same DN concentration in both MSN-DN experiments, as there was a slight variation in DN loading between the differently functionalized particles (0.28 and 0.19 wt% for anti-B220 MSN-DN and 0.3 wt% for anti-HuCD9 MSN-DN). This relatively low DN loading was used in order to avoid premature DN release. A fast release of a fraction of DN upon exposure to serum was observed for higher DN loadings, and this prematurely released fraction increased with higher DN loading (data not shown). Importantly, only the treatment with anti-B220 MSN-DN significantly delayed leukemia onset with a median latency of 160 days compared to 19–22 days in the different controls (p < 0.0001) (Fig. 3B). Delay in leukemia development correlated with reduced proportion of engrafted GFP^+^B220^+^ LSCs in the mice (11.6% and 23.2% after treatment with anti-B220 MSN-DN versus free DN, respectively, 24 hours before transplantation)(Fig. 3C, Supplementary Fig. S4). These results highlight efficient LSC killing by the anti-B220 targeted MSN-DN particles.

## Discussion

There is urgent need to develop innovative therapeutic concepts for patients with AML as still the majority of patients dies of this disease despite intensive polychemotherapy and allogeneic transplantation today^4^. As AML is propagated by LSCs, there is the rationale to develop therapies, which are able to preferentially target LSCs. One approach is to use functionalized nanoparticle based approaches, which are linked to antibodies recognizing antigens expressed on LSCs. Following this concept, we developed zwitterionic MSNs, tagged with anti-B220 antibodies, thereby recognizing and binding to B220^+^ LSCs in a murine model of CALM-AF10 positive AML^9^. Nanoparticle based approaches for elimination of cancer stem cells have been investigated before, but focused so far mainly on solid cancer using largely different delivery systems^14–16^. Reports on targeted approaches for hematopoietic malignancies are scarce. Recently, nanoparticles were used carrying the selective Aurora kinase B inhibitor AZD2811 to target HL-60 and MOLM-13 AML cell lines. These nanoparticles showed improved efficacy compared AZD1152 alone, in particular when combined with Ara-C^17^. In another report, light-inducible polymeric retinoic acid (RA)-containing nanoparticles with the capacity to accumulate in the cytoplasm of leukemia cells were used. Upon exposure to blue/UV light they released RA accompanied by differentiation of cells^18^. However, both approaches did not use functionalized nanoparticles targeting LSCs. In contrast to this, Jiang *et al*. applied a targeted system, using FLT3 ligand-conjugated nanoparticles carrying the tumor suppressor miR-150. With this approach the authors documented selective killing of FLT3-positive AML cells, confirming the potential of targeting nanoparticle systems^19^. Nanoparticle based concepts using surface proteins which are internalized after ligand binding as target such as the folate or the EGF receptor were already successfully applied in preclinical models of solid cancers^20–22^.

In our approach we confirmed specificity of drug delivery by performing B220 blocking experiments and by using nanoparticles linked to an anti-CD9 antibody as control. Furthermore, we demonstrated a significantly higher toxicity of anti-B220 MSN-DN on B220^+^ AML LSCs compared to B220^-^ AML LSCs, by this approach comparing cells with highly similar morphology, metabolic state and nature, but discriminated by B220 antigen expression (Supplementary Fig. S5). These proof-of-principle experiments demonstrate that it is feasible to tag MSNs with antibodies and to use these nanoparticles in a targeted approach in a leukemia model. Future *in vivo* experiments will have to show that also in an *in vivo* setting anti-B220 MSN-DN have no off-target toxicity to normal B220-negative hematopoietic bystander cells such as T – cells. The advantage of this system is that it allows rapid exchange of the covalently bound antibody by alternative antibodies binding to a different surface protein on leukemic stem cells. Recently, several antigens were identified, which are preferentially expressed on human leukemic stem cells in AML such as TIM3, CD96 or CD123^23^. However, most of these antigens are not actively internalized by the cells after ligand binding. This might reduce efficacy of functionalized MSNs. In our model we selected B220 because expression of this antigen distinguishes LSCs from normal HSCs^8^. Also B220 has the potential disadvantage that it is not internalized after antibody binding. Preferential toxicity of the B220-tagged MSNs in our system is most likely caused by MSN enrichment on the cell membrane of B220-positive cells followed by passive uptake, leading to the observed target cell specificity. In contrast to TIM3 and CD96, the IL-3 receptor or CD123 is actively internalized after antibody binding and is therefore a promising alternative candidate for nanoparticle approaches targeting LSCs. Thus, one key step in future experiments will be to adapt our system by moving from the B220 target to the targets which are actively incorporated into the cell. Another approach will be to tag MSNs to peptides, which bind and inhibit ligands, which are known to trigger cancer growth when activated. One example for this is CXCR4, which plays a major growth promoting role for AML LSCs, but also for a variety of B – cell lymphomas such as Waldenström’s macroglobulinemia (WM)^24–26^. We recently identified a naturally occurring anti-CXCR4 peptide EPI-X4, which significantly impairs engraftment of patient derived AML LSCs and WM cell lines^27,28^ (data not shown). We started to tag MSNs with EPI-X4 and optimized peptide derivatives of EPI-X4 with higher activity and increased stability. The anti-leukemic efficacy of these functionalized MSNs will be tested in the human system on AML and WM cell lines, but also on primary AML patient samples, which express CXCR4 in the majority of cases^29^. Importantly, EPI-X4 and its derivatives crossreact to murine Cxcr4, so that the potential of these MSNs can be tested in murine AML and lymphoma models.

In summary, our data indicate that functionalized antibody-tagged MSNs are able to target LSCs *in vitro* and efficiently kill AML LSCs at DN concentration levels well below those needed by free DN. The efficient *in vitro* AML LSC killing is also evident by a strongly prolonged survival of mice into which the pre-treated LSC were injected. Further optimization of the MSNs by targeting them to antigens, which are actively incorporated after ligand binding or by tagging them with peptides, which interfere with cancer growth promoting ligands will be key steps in the future. Beside this, modifications of the nanoparticles themselves by optimization of both the surface chemistry, the size and the shape of the particles will be a prerequisite to achieve longer half – lives and prolonged blood circulation times of the particles, a key prerequisite for their successful clinical use^30^.

## Methods

### Characterization of particles

Based on nitrogen sorption measurements on a Quadrasorb SI (Quantachrome) the BET surface area and NL-DFT (equilibrium kernel for silica with cylindrical pores) pore size distribution of the particles was calculated. Particle morphology and particle size was visualized by transmission electron microscopy on a TEM 1400 (Jeol). Dynamic light scattering was used to measure the hydrodynamic diameter as well as the zeta potential of the particles in a dispersion of particles (0.1 mg/mL) in 2-[4-(2-hydroxyethyl)-1-piperazinyl]-ethanesulfonic acid (HEPES) buffer solution (25 mM, pH 7.2) from Merck KGaA (Darmstadt, Germany) on a Zetasizer Nano-ZS ZEN 3600 (Malvern Instruments).

### Particle synthesis

Particle synthesis was performed according to the literature^11^. Briefly, cetyltrimethylammonium bromide (CTAB) was added to a solution of water (53.5 mol), caustic soda solution (4.6 mmol) and methanol (20.0 mol), followed by a mixture of tetramethoxysilane (TMOS) and 3-aminopropyltrimethoxysilane (APTMS). The reaction mixture was continuously stirred for 24 hours at room temperature. All above mentioned chemicals were purchased from Sigma-Aldrich Chemie GmbH (Munich, Germany). The particles were separated via centrifugation and surfactant was removed by three times extraction with ammonium nitrate in ethanol (6.0 g/L) for 1 hour in an ultrasonic bath. The particles were washed two times with ethanol and dried at 60 °C overnight.

### Functionalization with succinic anhydride

Particles were dried prior to functionalization with succinic anhydride in vacuum for 3 hours at 80 °C. Ethanol was dried with sodium under reflux conditions and then stored under inert atmosphere over a 3 Å molecular sieve. The dried particles were dispersed in dry ethanol (15.0 mg/mL) supplemented with succinic anhydride from Merck KGaA (Darmstadt, Germany) (0.4 µmol/mg) and rotated for 24 hours. Afterwards the particles were washed three times with ethanol and dried overnight.

### Fluorescence labelling

For intracellular detection the particles were labelled with the fluorescent dye ATTO 594 (ATTO-TEC GmbH, Siegen, Germany). For this particles were dispersed in HEPES buffer solution (20.0 mg/mL) (25 mM; pH 7.2) and ATTO 594 N-hydroxysuccinimide (NHS)-ester (1.3 nmol/mg) was added to the dispersion. After 1 hour of rotation at room temperature the particles were separated, washed two times with ethanol and dried for 24 hours at 60 °C. Ammonium nitrate, methanol, ethanol, potassium chloride and sodium hydroxide were obtained from Merck KGaA (Darmstadt, Germany).

### Neutralization of daunorubicin hydrochloride and loading on the particles

Daunorubicin hydrochloride (CHEMOS GmbH, Regenstauf, Germany) was neutralized with triethylamine according to the procedure previously described^31^. Daunorubicin hydrochloride (7.0 µmol) was dissolved in water (27.8 µmol) and triethylamine (8.4 µmol) was added. After 10 min of rotation, the neutralized form of daunorubicin was extracted with dichloromethane. The dichloromethane was removed in vacuum and the daunorubicin was vacuum dried for 24 hours. To remove adsorbed water from the pores of the particles, the particles were dried for 3 hours at 80 °C and afterwards dispersed in daunorubicin (Thermo Fisher Scientific Inc., Waltham, USA) (7.0 µg/mg) containing dichloromethane. Dichloromethane was dried over 4 Å molecular sieve. All other chemicals were used as received without further purification. After 24 hours of rotation at room temperature the particles were centrifuged and washed three times with dichloromethane before dried in vacuum for 24 hours at room temperature. The amount of daunorubicin on the particles was calculated based on UV/vis spectroscopic analysis on a SPECORD^®^ 50 (Analytik Jena) of the starting solution and the supernatant as well as the washing waters (λ = 499 nm) (calibration curve: y = 0.0101 mL/nmol ∙ x + 0.0375; R² = 0.9984; n = 3).

### Antibody functionalization

To conjugate the antibody, the carboxyl groups of the antibody anti-human/mouse CD45R (B220) respectively anti-human CD9 (20 µg/mL) was activated with N-(3-Dimethylaminopropyl)-N′-ethylcarbodiimide (EDC) (19.2 µmol/mL) and NHS (19.9 µmol/mL) in HEPES buffer solution (25.0 mM; pH 7.2) for 30 min at room temperature. The particles were dispersed in HEPES buffer solution (2 mg/mL), added to the solution with the activated antibody and rotated for further 90 min. Afterwards the particles were separated from the conjugation mixture, washed once with HEPES buffer solution before dispersed in DMEM supplemented with 15% FBS, 1% penicillin-streptomycin and 10 ng/ml RMIL310. The residual amount of daunorubicin on the particles was calculated based on ultra-violet (UV)/visible spectroscopic analysis of the supernatant and washing water (λ = 481 nm) (calibration curve: y = 0.0122 mL/nmol ∙ x – 0.0072; R² = 0.9995; n = 3).

### Cell culture

CALM-AF10 positive murine AML cells were derived from single-cell-sorted B220^+^/Mac1^−^ cells, which were shown to have LSC characteristics, reflected by their ability to cause AML in serially transplanted mice (data not shown)^9^. These sorted B220^+^/Mac1^−^ single cells were expanded *in vitro* (B220^+^ AML LSCs) and used for experiments. B220-negative AML LSCs, also proven to be able to cause leukemia in serial transplanted mice, were derived from the bone marrow of leukemic mice transplanted with bone marrow (BM) retrovirally engineered to overexpress the homeobox gene Cdx2 as previously published^32,33^. These cells were growing immortally as a homogenous B220-negative cell population *in vitro*. Before these two LSC populations were used for experiments, B220 positivity or negativity was confirmed by FACS. Cells were cultured in 15% fetal bovine serum (FBS) in 1X dulbecco’s modified eagle medium (DMEM) containing 1% penicillin-streptomycin solution and 10 ng/ml recombinant mouse interleukin3 (RMIL310)(Thermo-Fischer Scientific (Waltham, MA USA).

### *In vitro* and *in vivo* experiments

All *in vitro* treatments were performed with anti-human/mouse CD45R (B220) (anti-B220) tagged MSNs (mesoporous silica nanoparticles with carboxyl functionalization) and anti-human CD9 tagged MSNs loaded with or without daunorubicin. Experiments were performed for 24 hours at 37 °C and 5% CO_2_. B220 antigen was blocked on the CALM-AF10 positive AML cells (B220^+^ murine AML LSCs) by incubation with the anti-human/mouse CD45R (B220) antibody Clone RA3-6B2, eBioscience Inc., San Diego, USA) for 4 hours. Cells were incubated with the anti-human/mouse CD45R (B220) tagged or anti-human CD9 tagged MSNs loaded with daunorubicin for 24 h. For all flow cytometry and microscopic analyses, cells were washed 1 time with 1 × dulbecco’s phosphate buffered saline (DPBS) from Thermo-Fischer Scientific (Waltham, MA USA) containing 0.01% Tween-20 from Sigma-Aldrich Co. LLC (St. Louis, MO) thereafter with 2 consecutive 1 × DPBS washes after incubation with MSNs.

For *in vivo* experiments, 2 × 10^5^ cells were plated *in vitro* and incubated for 24 h as described above. Cells left from the 2 × 10^5^ cells at day 0 after treatment (2 × 10^5^ day 0 equivalent) together were injected with 1.25 × 10^6^ non-irradiated helper cells intravenously in lethally irradiated (1200 cGy) C57BL/6 mice. All animal experiments were performed in compliance with the ETS123 (European Convention for the Protection of Vertebrate Animals used for Experimental and other Scientific Purposes) and guideline 2010/62/EU and were approved by the Regierungspräsidium Tuebingen, Germany.

### Flow cytometry and analysis

All flow cytometry experiments were performed on a BD (Becton Dickinson GmbH, Bioscience, NJ, USA) LSR Fortessa cell analyzer and sorting for the B220^+^Mac1^−^ cells were performed on a BD FACS ARIA II. Flow cytometry analysis was performed using BD FACS Diva v8.0.1 and analyzed using Microsoft Excel 2013 and GraphPad Prism 6.0.

### Statistics

Data were analyzed using appropriate testing such as 2-tailed paired student’s t-test, Wilcoxon-mann-whitney-test and area under the curve (AUC) analysis by GraphPad Prism version 6.0 or with Microsoft Excel 2013 for linear regression fitting and trend line analysis.

## Electronic supplementary material

Supplementary Information
