# Supplementary material for: Targeting murine leukemic stem cells by antibody functionalized mesoporous silica nanoparticles
Source: Sci Rep. 2018 Jan 17;8:989. doi: 10.1038/s41598-017-18932-4 (PMC5772366; doi:10.1038/s41598-017-18932-4)
Supplement: Supplementary file 1 — Supplementary Information [file 41598_2017_18932_MOESM1_ESM.pdf]

Supplementary Information

Targeting murine leukemic stem cells by antibody functionalized mesoporous silica nanoparticles

Tamoghna Mandal[1#], Michaela Beck[2#], Nicole Kirsten[1], Mika Lindén[2*], Christian Buske[1*]

1. Institute for Experimental Cancer Research, CCC and University Hospital of Ulm, Albert-Einstein-Allee 11, 89081 Ulm, Germany.
2. Institute for Inorganic Chemistry II, University of Ulm, Albert-Einstein-Allee 11, 89081 Ulm, Germany

[#]Authors have contributed equally

***Corresponding authors:**

Christian Buske: Institute of Experimental Cancer Research, CCC and University Hospital of Ulm, Germany. Phone: +49 731 50065-888; Fax: +49 731 50065 802; E-mail: christian.buske@uni-ulm.de

Mika Lindén: University of Ulm, Institute of Inorganic Chemistry II, Albert-Einstein-Allee 11, 89081 Ulm, Germany. Phone: +49 731 5022 731; Fax: +49 731 5022 733; E-mail: mika.linden@uni-ulm.de

Supplementary figure Legends

Supplementary figure S1: Confocal fluorescence microscopy demonstrating intake of anti-B220 tagged MSN particles into $B220^+$ AML LSCs in contrast to the $B220^-$ AML LSCs. Nuclei were stained with DAPI (Blue), GFP (Green) is retrovirally expressed by the cells and ATTO 594 (Red) was covalently linked to MSNs. Particle concentration used was 50 µg/mL. One representative image from three (n=3) independently performed experiments. Magnification is 200X with corresponding 50µm scale bars shown.

Supplementary figure S2: Cumulative analysis of PROBIT converted values (median cell death %) for anti-B220 tagged MSN-DN and free DN on $B220^+$ murine CALM-AF10 positive (CA10) AML LSCs with/without blocking with the anti-B220 antibody. All of the values are calculated from three (n=3) independently performed experiments. Respective treatments are shown in the legend. Linear trendline and R^2 values for the respective data points are indicated.

Supplementary figure S3: Representative FACS staining of $B220^+GFP^+$ LSCs for co-expression of CD9 and B220 antigen. Proportions of populations are indicated (n= 5).

Supplementary figure S4: Dot plots of murine bone marrow (n=3) surviving after treatment with anti-B220 MSN-DN. Proportions of leukemic GFP^+B220^+ LSCs and non-transduced healthy GFP^- $B220^+$ cells are indicated.

Supplementary figure S5: Bar graph representing cell death of $B220^+$ AML LSCs compared to $B220^-$ AML LSCs after treatment with anti-B220 MSN-DN and appropriate controls for 24 h (n=3)($p<0.01$).

Supplementary figure S1

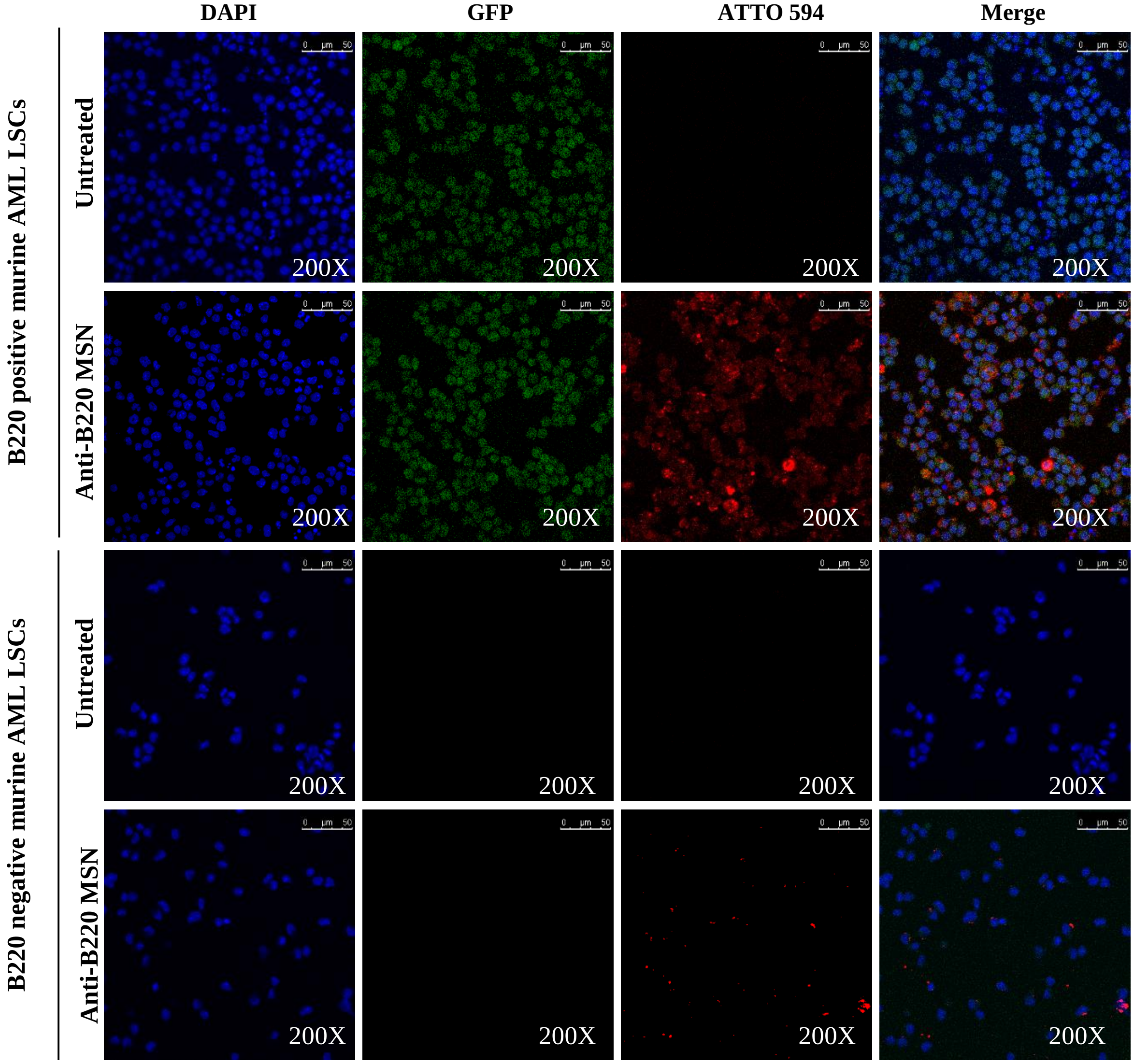

Supplementary figure S2

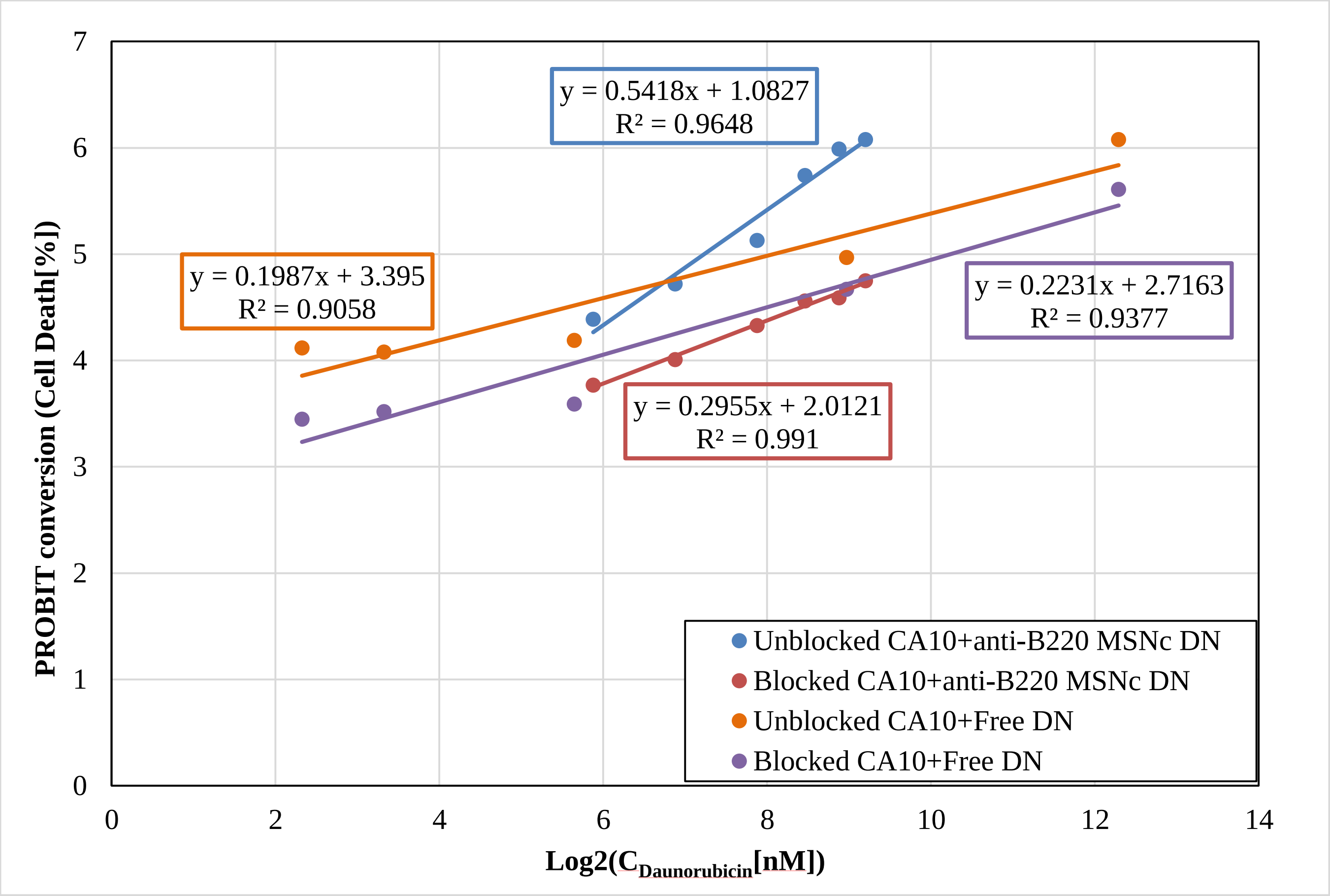

Supplementary figure S3

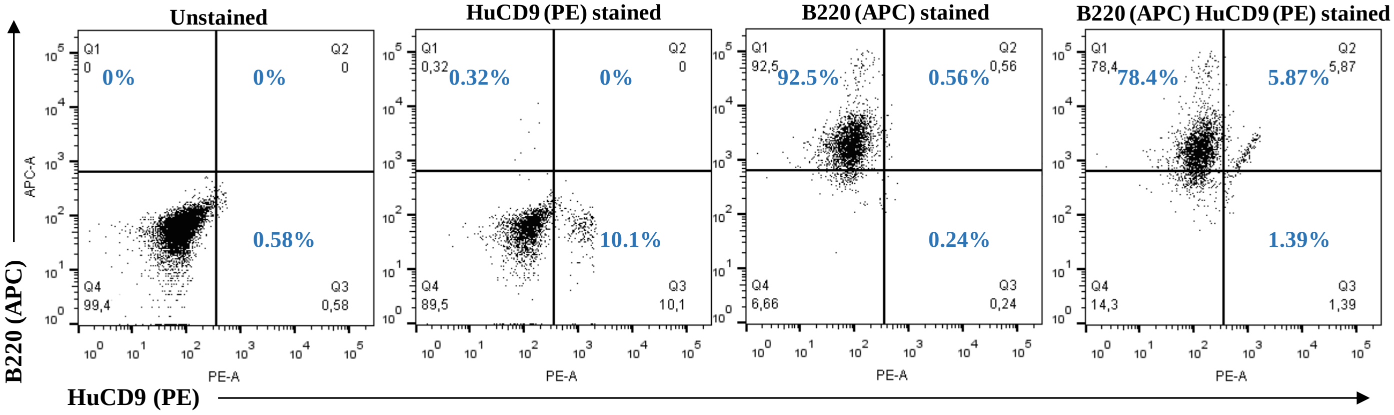

Supplementary figure S4

Healthy Mice

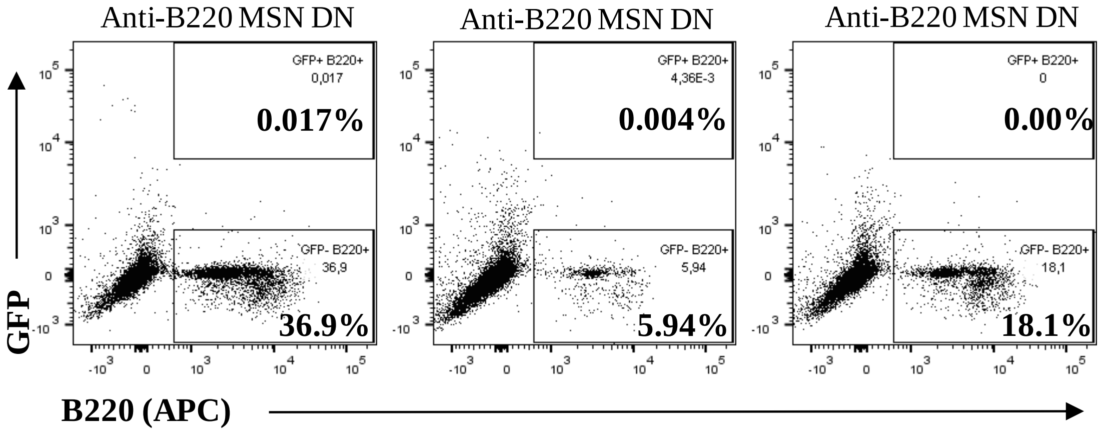

Supplementary figure S5

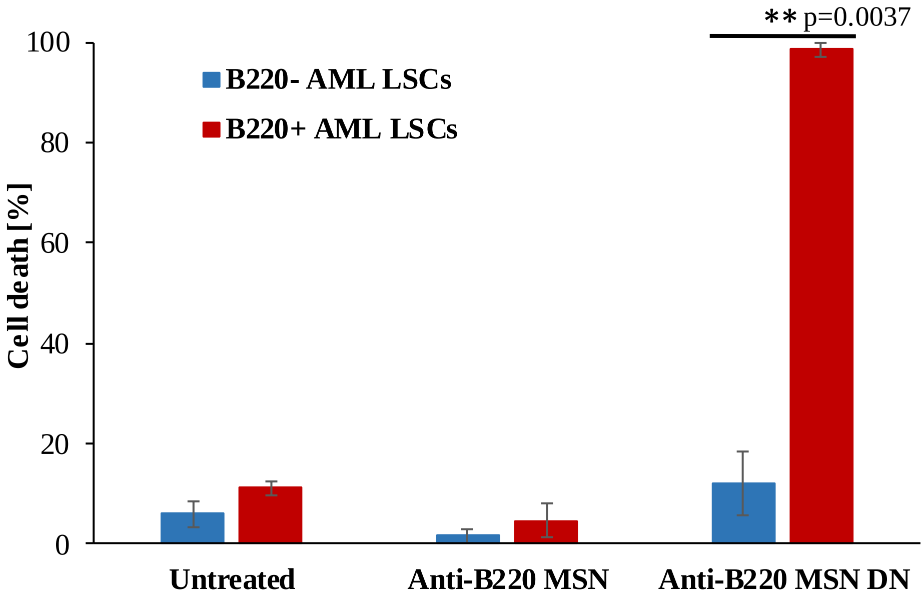

Supplementary Tables

Supplementary table S1: **Particle characterization**

Particle characterization with nitrogen sorption analysis, dynamic light scattering (DLS) and zeta potential measurements of amino and succinic anhydride functionalized particles. MSNc denotes the MSN after succinic anhydride treatment.

	$n_{succinic\ anhydride}/m_{particle}$ [mmol/g]	$d_{hydrodynamic\ diameter}$ [nm]	ζ-Pot [mV]	S_{BET} [m²/g]	d_{DFT} [nm]	d_{TEM} [nm]
MSN	0	390	-9	860	3.4	190
MSNc	0.4	250	-27	710	3.3	-

Zeta potential measurement and dynamic light scattering were performed in HEPES buffer solution (pH 7.2; 25 mM)

Supplementary table S2: Daunorubicin loading on the particles used in different experiments

Experimental details		$m_{dauno}/m_{particle}$ [µg/mg]	$n_{dauno}/m_{particle}$ [nmol/mg]	$m_{particles}$ [µg] per mouse	n_{dauno} [pmol]
Blocked versus unblocked B220^{+} AML LSCs anti-human/mouse B220 MSN-DN (Figure 2C)		3.1	5.9	-	-
Particles functionalized with different antibodies (Figure 2E)	anti-human/mouse B220 MSN-DN	4.3	8.1	-	-
	anti-human CD9 MSN-DN	3.2	6	-	-
Mouse experiment Exp1(Figure 3B)	anti-human/mouse B220 MSN-DN	2.8	5.3	100	530
	anti-human CD9 MSN-DN	3	5.8	100	580
	Free daunorubicin (13 µM)	-	-	-	500
Mouse experiment Exp2(Figure 3B)	anti-human/mouse B220 MSN-DN	1.9	3.7	157	576
	Free daunorubicin (13 µM)	-	-	-	572
B220+ vs B220- AML LSCs anti-human/mouse B220 MSN-DN (Supplementary Figure S5)		4.2 (average)	7.9 (average)	-	-

Supplementary table S3: Experimental set up for *in vivo* experiments

**Experiment 1/2 *In vitro* treatment for 24 hours**	**Day 0 Cell counts (Total no. of cells/ml)**	**Cell counts after 24 hour treatment**	
		**Experiment 1 (Total no. of cells/mice)**	**Experiment 2 (Total no. of cells/mice)**
Untreated	$1x10^5$	$2x10^5$	$2.12x10^5$
Anti-B220 MSN	$1x10^5$	$1.98x10^5$	-
Free Daunorubicin	$1x10^5$	$1.6x10^5$	$1.5x10^5$
Anti-HuCD9 MSN-DN	$1x10^5$	$1.76x10^5$	-
Anti-B220 MSN-DN	$1x10^5$	$1.52x10^5$	$1.08x10^5$

Summary of cells used in the *in vivo* experiments (Figure 3A) giving the day 0 equivalent and the cell number injected after 24h incubation for the different experimental arms.
